# Economic evaluations of vaccines in Canada: a scoping review

**DOI:** 10.1186/s12962-017-0069-4

**Published:** 2017-05-05

**Authors:** Ellen R. S. Rafferty, Heather L. Gagnon, Marwa Farag, Cheryl L. Waldner

**Affiliations:** 1 School of Public Health, University of Saskatchewan, Saskatoon, SK S7N 5A2 Canada; 2 Alberta Health Services, Calgary, AB T2N 2T9 Canada; 3 Western College of Veterinary Medicine, University of Saskatchewan, Saskatoon, SK S7N 5A2 Canada

**Keywords:** Economic evaluation, Review, Vaccines, Canada, Cost-effectiveness, Scoping review

## Abstract

**Background:**

This study aims to summarise and describe the evolution of published economic evaluations of vaccines in Canada, thereby outlining the current state of this expanding and meaningful research.

**Methods:**

Using Arksey and O’Malley’s scoping review framework we assembled relevant research from both academic and grey literature. Following abstract and full-text review we identified 60 articles to be included in the final analysis.

**Results:**

We found that since 1988 there has been a steady increase in the number of economic evaluations on vaccines in Canada. Many of these studies focus on the more recently licensed vaccines, such as influenza (16.7%), human papillomavirus (15.0%) and pneumococcal disease (15.0%). Since 2010 economic evaluations of vaccines have shown increased adherence to economic evaluation guidelines (OR = 4.6, CI 1.33, 18.7), suggesting there has been improvement in the consistency and transparency of these studies. However, there remains room for improvement, for instance, we found evidence that studies who stated a conflict of interest are more likely to assert the vaccine of interest was cost-effective (OR = 7.4; CI 1.04, 17.8). Furthermore, most reports use static models that do not consider herd immunity, and only a few evaluate vaccines post-implementation (ex-post) and traveller’s vaccinations.

**Conclusion:**

Researchers should examine identified research gaps and continue to improve standardization and transparency when reporting to ensure economic evaluations of vaccines best meet the needs of policy-makers, other researchers and the public.

**Electronic supplementary material:**

The online version of this article (doi:10.1186/s12962-017-0069-4) contains supplementary material, which is available to authorized users.

## Background

Vaccination is lauded as one of public health’s most significant achievements, contributing to improvements in morbidity, mortality and quality of life worldwide [1, 2]. Originally, one of the major advantages of vaccination as a public health intervention was their low cost, as many of the classic Expanded Program on Immunization vaccines only cost a few cents per dose and were cost-saving to the healthcare system [1, 3]. However, in recent years more technologically complicated vaccines and more strict licensing regulations for vaccine safety and efficacy have led to more expensive vaccine development and manufacturing, which raises the question of vaccine cost-effectiveness [3].

With ongoing debates in many countries concerning the implementation and funding of these new vaccines, especially within the context of growing financial strain on healthcare systems, economic evaluations are becoming increasingly important to policy-makers [3]. Economic evaluations can improve the quality and consistency of decision making by providing a systematic way of comparing whether a specific vaccination program should be adopted compared with doing nothing or implementing an alternative intervention (e.g. treatment, other vaccines). Economic evaluations can also help evaluate scheduling and target population(s) [4, 5].

The number of vaccine-related economic evaluations has substantially increased in the last few years. However, we have very little knowledge of the coverage and quality of this research, as well as the potential gaps and limitations of these studies [6]. Systematic reviews on economic evaluations of immunizations generally focus on the results from a particular vaccine; recent examples include varicella [7] and influenza [8]. Meanwhile, many countries have yet to review and synthesize their own research in this area. One of the few examples is Spain, who recently released two comprehensive systematic reviews on Spanish economic evaluations of vaccinations completed between 1990 and 2012 [9, 10].

In comparison, researchers in Canada have never fully synthesized the extent and characteristics of research on economic evaluations of vaccines in Canada. As the number of licensed vaccines in Canada and the number of economic evaluations on those vaccines continue to increase, it is essential that Canada begins to critically analyse and summarise this work [6]. This research is important to ensure improvement in the usefulness, quality and applicability of vaccine-related economic evaluations and the decisions they inform. Furthermore, as countries produce similar evidence, comparisons of reporting, methods and results between countries could contribute to the international discussion on the major gaps in the literature and the quality, standardization and transparency of these studies.

This study aims to systematically gather, review and summarise Canadian economic evaluations on vaccines using a scoping review methodology, with the goal of describing general trends and gaps in the literature.

## Methods

### Scoping review methodology

We based our methodology on Arksey and O’Malley’s [11] five step framework for conducting a scoping review, including (1) forming the research question, (2) identifying relevant studies, (3) study selection, (4) charting the data and (5) summarising and reporting the results. Each section is described in more detail below. Scoping reviews are designed to summarise rich and complex areas of research that have not be synthesized in the past [11]. A scoping review also offers policy-makers easily accessible and comprehensive cost-effectiveness information and evidence regarding vaccines. Moreover, a review helps identify gaps in the vaccine economic evaluation literature and aids policy-makers spend their limited research funds more effectively and efficiently. We chose to conduct a scoping review rather than a systematic review because it allowed us to methodologically examine the breadth and depth of the work on economic evaluations of vaccines in a Canada, a highly multi-disciplinary area, while allowing for more flexibility than a systematic review [12]. The aims of our study were more consistent with the goals of a scoping review (e.g. to map the current state of the literature and summarize the breadth and depth of the research) in comparison to the goals of a systematic review (e.g. to summarize the evidence on the effectiveness of an intervention or treatment) [12]. The scoping review methodology allowed us to summarize a wide range of evidence including both grey (e.g. government reports) and peer-reviewed literature. Furthermore, rather than having a very specific and narrow research question, we asked a broad research question that encompassed all types of diseases, populations (target vs. universal) and interventions (i.e. types of vaccine) [12].

### Identifying the research question

To further focus our research question—“What has been published regarding economic evaluations of vaccines in Canada?”—we decided to only consider active immunization, vaccines that impact human health, and studies that focused on a Canadian population. To ensure comparability we chose to include only full economic evaluations, which we defined based on Drummond et al., and therefore, partial economic evaluations such as cost-outcome description studies and cost-minimization studies were not included [5, 13]. Furthermore, to ensure the comprehensiveness of the review, we included both peer-reviewed and grey literature in our search. Only English-language articles were part of the final analysis.

### Identifying relevant studies

To identify peer-reviewed articles we searched five databases relevant to both public health and economic evaluations: Embase, Medline, Global Health, Cochrane Library—specifically the Health Technology Assessment Database—and the NHS Economic Evaluation Database. No limitations were placed on the date and all databases were searched up until March 17th, 2015. We used a report-based expansion strategy centred on three essential keywords (‘immunization’ AND ‘economic evaluation’ AND ‘Canada’). See Additional file 1 for a full list of search terms used. After title, abstract and full-text review, the reference lists of all included articles were searched for relevant citations. We validated each database search for its efficiency and accuracy using ten articles that the reviewers identified as being highly relevant to the topic area. We compared the ten articles to the citations we identified during each database search.

To identify grey literature we searched both the ProQuest Dissertation and Theses database along with key organizational websites related to health technology assessment, vaccination and economics in Canada, such as Canadian Agency for Drugs and Technologies in Health’s (CADTH), the Health Quality Council from multiple provinces and Institute of Health Economics. We chose these organizations based on advice from a health sciences librarian, the opinion of experts in the field and the CADTH grey literature checklist [14]. In total we searched 25 potentially relevant websites and organizations.

### Study selection

All eligible articles were imported into Microsoft Access 2010 for relevance screening and duplicates were removed. Independently, two reviewers evaluated the titles, abstracts and full-text of peer-reviewed articles by answering ‘yes,’ ‘no,’ or ‘unsure’ to each of the following questions:

1. Was the research conducted on a Canadian population? (i.e. presented Canadian-specific data or results?)
2. Was a full economic evaluation conducted (measured both costs and benefits)?
3. Did the topic area relate to active vaccination or immunizations? (e.g. different types of vaccines, different schedules)?
4. Is a full-text available?
5. Is the article in English?
6. Were the results relevant to human health?

During title, abstract and full-text screening of an article if the answer to any of the above questions was ‘no’ then the article was excluded; otherwise, the article was included in the next stage of analysis. We calculated Cohen’s Kappa Coefficient at both the title, abstract and full-text screening stages to measure agreement between the two reviewers [15]. The reviewers resolved any disagreements through discussion and consensus. One reviewer screened the grey literature for inclusion or exclusion using the same process outlined above; however, if the first reviewer was uncertain about the inclusion of an article it was given to the second reviewer. All the grey literature articles included in the final analysis were read by both reviewers.

### Charting the data

Following full-text screening, the two reviewers charted each study chosen for inclusion using a standardized form designed to gather common and comparable information on each study. Data extracted included year of publication, region, targeted disease, type of economic evaluation, modelling type, herd immunity, target population, study perspective, time horizon, type of sensitivity analysis, comparator, the consideration of equity issues, general cost-effectiveness findings and stated conflict of interest(s). These variables were chosen based on CADTH’s Guidelines for the Economic Evaluation of Health Technologies: Canada [16] and the World Health Organization’s (WHO) Guide for Standardization of Economic Evaluations of Immunization Programmes [3] that outline information to include in an economic evaluation. The main focus of our scoping review was to summarize the range of evidence and identify any gaps in the literature, rather than compare the results of the economic evaluation; therefore, we did not to compare the articles based on quality. We charted each study’s adherence to these guidelines, and evaluated each study on whether they adequately (according to guideline protocols) presented six key principles that are considered essential for a well conducted economic evaluation. These six key principles included: time horizon, perspective, comparator, model-type and choice of economic evaluation (including justification for choice of model and economic evaluation), and if uncertainty in the results was fully accounted for through sensitivity analysis. This analysis gives policy-makers not only an idea of how economic evaluations are generally being conducted and presented in Canada, but also the effectiveness of their guidelines to inform research and whether the guidelines could be applied consistently across articles.

### Summarising and reporting the results

Following data extraction we used thematic analysis and descriptive statistics to analyze general trends and patterns in the data. We used Chi square tests to ascertain whether there was an association between stated conflicts of interests (i.e. at least one authors reported they were either currently working for the pharmaceutical industry or currently receiving funding) and their final cost-effectiveness recommendations (i.e. concluded the vaccine of interest was cost-effective or not). Furthermore, we studied the association between the publication year (i.e. <2010 or ≥2010) and the studies adherence to the six key principles of an economic evaluation discussed in the WHO and CADTH guidelines (i.e. adhered to guidelines or not). We also examined the association between publication year (i.e. <2010 or ≥2010) and choice of modelling type (i.e. static vs. dynamic). We chose 2010 as the cut off year as it provided adequate time after the publication of the WHO [3] and CADTH [16] guidelines for authors to incorporate the recommendations in their research. Finally, we calculated the number of studies that took place prior to licensure of the vaccine in Canada, between licensure and the implementation of the vaccination program being studied, and those that occurred after the implementation of the vaccine in the region considered in the study. We were particularly interested in economic evaluations that aimed to re-evaluate the cost-effectiveness of the vaccine post-implementation and therefore included retrospective data (ex-post).

## Results

**Fig. 1:**
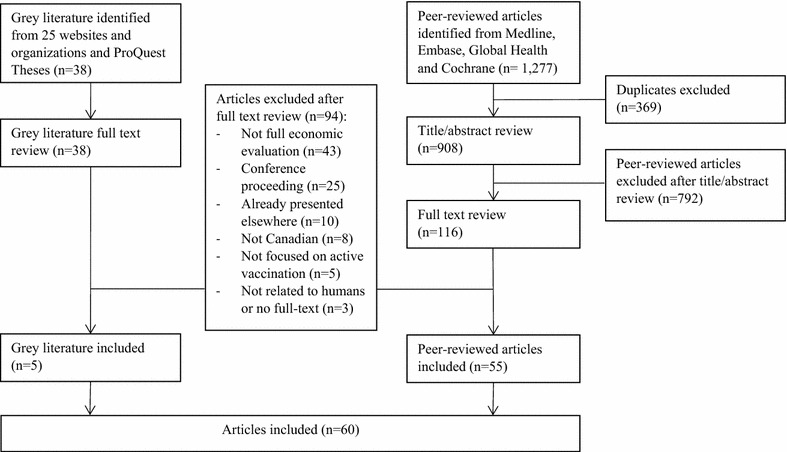
Flowchart for the identification and selection of studies included in the scoping review

Following the database search and the removal of duplicates, 908 peer-reviewed articles remained to be screened. Based on title and abstract screening we eliminated 792 articles, leaving 116 articles for full-text review, of which 55 were included in the final analysis (Fig. 1). All ten ‘original’ studies chosen as being highly relevant to the research and used to validate the search methods were identified in each of the database searches. There was substantial agreement between the two reviewers; Cohen’s Kappa Coefficient was 0.81 for the title and abstract screening and 0.85 for the full-text screening. The reviewers reached consensus in all cases of disagreement.

Following the grey literature search and title screening we retrieved 38 potentially relevant studies and dissertations from 25 websites and the ProQuest Theses database, of which five were retained in the final analysis. Sixty peer-reviewed and grey-literature studies were included in the final scoping review analysis (Fig. 1).

### Summary of studies included in the review

**Fig. 2:**
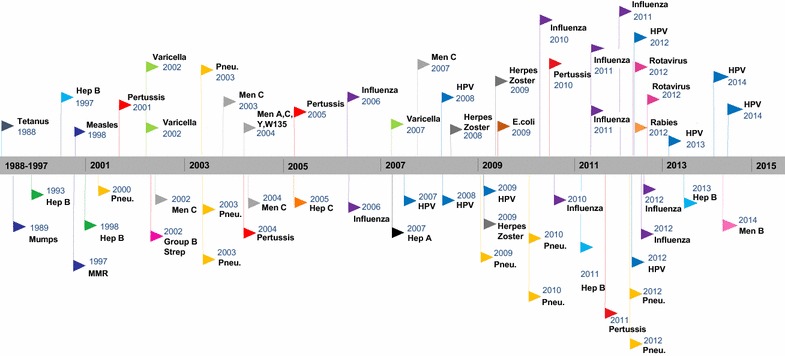
Timeline of Canadian economic evaluations of vaccines *All the flag colours in the above figure represent a different disease

The number of articles by year of publication, region, disease, type of economic evaluation, modelling type, consideration of herd immunity, perspective, time horizon and sensitivity analysis were summarized (Table 1). The number of economic evaluations of vaccines steadily increased between 1988 and March 2015; 66.7% of economic evaluations were published within the last ten years (i.e. since 2005) (Fig. 2). Most studies focused on Canada in general (58.3%), with Quebec (13.3%), Ontario (10.0%) and British Columbia (8.3%) producing the most province-specific vaccine economic evaluations. Economic evaluation of vaccines in Canada considered 20 different diseases including, from most common to least common—influenza (pandemic-2 and seasonal-8) [17–26], human papillomavirus (HPV) [27–35], pneumococcal [36–44], pertussis [45–49], meningitis A, C, Y and/or W135 [50–54], hepatitis B [55–59], varicella [60–62], measles, mumps and/or rubella [63–65], herpes zoster [66, 67], rotavirus [68, 69], rabies [70], hepatitis A [71], tetanus [72], hepatitis C [73], meningitis B [74], group B streptococcus [75], and Escherichia coli [76] (Fig. 2).

**Table 1:** Summary descriptive statistics and variable frequency for 60 vaccine economic evaluations

Variable	Number of studies included	Percentage of total (n = 60) (%)
Source		
Peer-reviewed	55	91.7
Grey-literature	5	8.3
Year of publication		
Before 1995	3	5.0
1995–2004	17	28.3
After 2005	40	66.7
Region		
Canada	35	58.3
Atlantic^a^	2	3.3
Quebec	8	13.3
Ontario	6	10.0
Manitoba	1	1.7
Saskatchewan	0	0.0
Alberta	3	5.0
British Columbia	5	8.3
Territories^b^	0	0.0
Disease		
Influenza	10	16.7
HPV	9	15.0
Pneumococcal	9	15.0
Pertussis	5	8.3
Meningoccocal A, C, Y or W135	5	8.3
Hepatitis B	5	5.0
Varicella	3	5.0
Measles, mumps or rubella	3	5.0
Herpes zoster	2	3.3
Rotavirus	2	3.3
Rabies	1	1.7
Hepatitis A	1	1.7
Tetanus	1	1.7
Hepatitis C	1	1.7
Meningoccocal B	1	1.7
Group B Streptococcus	1	1.7
* Escherichia coli*	1	1.7
Type of economic evaluation		
Cost-utility	37	50.0
Cost-effectiveness	30	40.5
Cost-benefit	7	9.5
Modelling type		
Simple tree	16	21.6
Static cohort	25	41.7
Dynamic cohort	7	11.7
Individual-based	5	8.3
No modelling (e.g. RCT, simple calc.)	7	11.7
Consideration herd immunity		
Yes	25	41.7
No	35	58.3
Time horizon		
≤1 year	7	11.7
>1–29 years	14	23.3
30–79	8	13.3
80+	25	41.6
Not stated	6	10.0
Perspective		
Individual/Familial	4	6.7
Healthcare pay	35	58.3
Public pay	10	16.7
Societal	28	46.7
Not stated	8	13.3
Sensitivity analysis		
Yes-deterministic	53	88.3
Yes-probabilistic	23	38.3
No	5	8.3
Alternative/comparator		
No vaccine	40	66.7
Different vaccine programs	15	25
Different types of vaccines	16	26.7
Conflict of interest		
Yes	28	46.7
No	32	53.5

^a^ Includes the provinces of New Brunswick, Nova Scotia, Prince Edward Island, and Newfoundland and Labrador

^b^ Includes the territories of Yukon, Northwest Territories, and Nunavut

The most popular type of economic evaluation used in Canadian studies was cost-utility analysis (50.0%); although, it was often combined with cost-effectiveness analysis. More than half of the studies used static modelling (e.g. simple tree or static cohort) (63.3%). Most studies used a life-time horizon (i.e. 80+ years) (41.6%), with a healthcare payer (58.3%) or societal perspective (46.7%), often combining the two perspectives. Researchers employed a wide range of comparators (Table 2). The vaccine of interest was compared to no vaccine in 66.7% of studies. The evaluation compared different ways of administering the vaccine program (e.g. different schedules or target populations) in 25.0% of studies. Different types of vaccines (brands or antigen formulations) for the same disease were compared in 26.7% of studies, with some studies employing more than one type of comparator. Most studies (86.7%) evaluated publically-funded vaccines that were included on at least one provincial immunization schedule (Table 2). Finally, only six studies in our analysis had a discussion of equity, which mainly focused on the fact that they were unable to address issues of equity within their analysis.

**Table 2:** The vaccine comparators and schedules used in each economic evaluation

Vaccine	Number of studies—no vaccine	Number of studies—type of vaccine	Number of studies—vaccine program^b^	Funding and target population^c^
*Escherichia coli*	1 (0/1)^a^	–	–	Private
Group B Streptococcus	1 (1/1)	–	–	Not yet available
Hepatitis A	–	1	–	Private
Hepatitis B	5 (3/5)	–	1	Public-universal
Hepatitis C	1 (1/1)	–	–	Not yet available
Herpes Zoster	2 (2/2)	–	–	Private
HPV	7 (7/7)	4	2	Public-universal
Influenza—(seasonal and H1N1)	6 (5/6)	1	4	Public-Universal/targeted
Measles or Mumps or MMR	2 (2/2)	–	3	Public-universal
Meningococcal A, C, Y, W135	2 (1/2)	2	1	Public-universal
Meningitis B	1 (0/1)	–	–	Public-targeted/private
Pertussis	1 (1/1)	2	2	Public-universal
Pneumococcal	4 (4/4)	4	1	Public-universal
Rabies	1 (1/1)	–	–	Private
Rotavirus	2 (2/2)	2	–	Public-universal
Tetanus	1 (1/1)	–	–	Public-universal
Varicella	3 (2/3)	–	1	Public-universal

^a^ Ratio represents the number of studies that found the vaccine was cost-effective compared to no vaccine

^b^ For example: schedule, booster dose, universal vs. targeted

^c^ Funding and target population for vaccines as of March 2015

### Factors associated with reporting practices and study findings

**Fig. 3:**
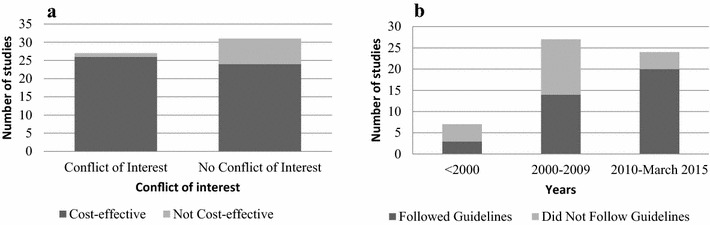
Associations between variables of interest.** a** Conflict of interest vs. cost-effectiveness.** b** Year of publication vs. adherence to guidelines

In reports where there was a stated conflict of interest, the authors were more likely to assert the vaccine of interest was cost-effective relative to the comparator (OR = 7.36; CI 1.04, 17.8; p value = 0.04) than in reports with no reported conflict. Furthermore, studies published from 2010 to 2015 were more likely to follow the six key principles in the WHO and CADTH reporting guidelines compared to those studies published before 2010 (OR = 4.58, CI 1.33, 18.7, p value = 0.01) (Fig. 3). However, there was no difference in the type of model employed before 2010 compared to 2010–2015 (OR = 3.05; CI 0.66, 16.8; p value = 0.16). All of the five individual-based models were published in the last five years [21, 28, 30, 31, 70].

Most studies (66.7%) were completed before the vaccine program of interest was implemented in Canada with 8.3% before the vaccine was licensed. One in four studies (25.0%) were completed after the vaccine program was implemented. Only five post-implementation studies (8.3%) were ex-post and evaluated past vaccination strategies [21, 22, 43, 48, 52].

## Discussion

In recent years, the number of economic evaluations on health interventions have increased in Canada and worldwide [16, 77]. The results of our scoping review, are consistent with these trends, with 50.0% of the economic evaluations of vaccines in Canada being produced in the last five years [16, 77]. There are many explanations for this increase in economic evaluations, including increasing pressures on the budgets of healthcare systems, as well as the advent of new, complex and more expensive vaccinations [77, 78]. Therefore, it is perhaps unsurprising that the influenza, pneumococcal and HPV vaccines represented almost 50.0% of the economic evaluations, as the immunization programs for these diseases, including the schedules, vaccine-type, administration techniques and target populations have all changed over the last few years. For instance, recently decision-makers have raised questions surrounding the cost-effectiveness of male HPV vaccination, using the modified live intranasal influenza vaccine in children between two and 17, and which pneumococcal vaccine (i.e. PCV13, PCV10 and PCV7) would give the best value for the money, particularly considering the impact of serotype replacement and otitis media [26, 28, 30, 31, 36, 38, 39, 44].

Most economic evaluations focussed on publically-funded vaccines that were part of at least one provincial immunization schedule. A very small proportion of the research was done on privately-available vaccines, such as travel (e.g. yellow fever, Japanese encephalitis) and workplace vaccines (e.g. rabies) [70, 76]. Furthermore, there are only a small amount of research on vaccines not yet available or licensed in Canada (e.g. chlamydia, herpes simplex), with some notable exceptions (i.e. hepatitis C and meningococcal B) [73, 74]. Anticipatory economic evaluations can provide essential information on the needs for additional research and investment.

In Canada, most of economic evaluations occur after the vaccine has been licensed but before the implementation of the vaccine program in the population (ex-ante). These ex-ante studies are important as they provide decision-makers with an estimate of the value of instituting a new vaccine and the costs and benefits of different vaccination programs (e.g., private, targeted, universal). However, ex-ante evaluations are difficult to perform as country-specific effectiveness and cost data is often lacking before implementation, and the ability to predict the vaccination impact, particularly herd effects, is limited [79]. For instance, prior to vaccine implementation it is difficult to predict the real market price of a vaccine, which can vary dramatically in space and time. Therefore, there is a need to evaluate whether or not existing vaccination programs are good value for their cost, especially because effectiveness and cost data can change drastically over time [79]. These ex-post studies allow researchers to validate estimates of costs and outcomes from pre-implementation evaluations, and potentially improve future studies [79].

However, we saw little evidence of ex-post economic evaluations. For instance, the costs and outcomes of the varicella vaccine ex-post have not been compared with the two inconclusive studies conducted ex-ante [68, 69]. In fact, only five studies were identified where the goal was to determine the efficiency of a mass immunization after its implementation in the population [21, 22, 43, 48, 52]. This lack of retrospective research has left many unanswered questions, such as ‘How good are Canadian economic evaluations at predicting the cost-effectiveness of vaccines and should these studies inform our policy-making?’ This gap in the literature may be partially due to a lack of industry willingness to fund ex-post studies or because of noted methodological limitations, including issues with estimating the ‘no program’ scenario, attributing decrease in disease to vaccination and predicting future benefits [79]. However, Newall et al. [79] has identified a variety of approaches that help address these issues, noting that as retrospective studies become more important and frequent, it would be beneficial to increase the guidance available to researchers undertaking such analyses. In the future, a clearer understanding of the accuracy of economic evaluation predictions could help researchers improve research techniques and potentially increase the efficiency of current vaccination programs.

Most of the studies in this review compared the vaccine of interest to no vaccination. While this is an essential initial comparison, once a vaccine is well-established, there are other questions that economic evaluations can help answer, including questions about vaccine programming and scheduling. In Canada, healthcare is primarily under provincial/territorial (not federal) jurisdiction and as a result there is a diversity of vaccine programs, technologies and schedules throughout the country. To date there have been no reported studies of which vaccination programs are most efficient or cost-effective, or whether these differences are justifiable due to the epidemiology and costs in different Canadian regions.

The use of modelling in economic evaluation of vaccines has increased in the last few years. Most studies continue to employ static models (e.g. decision tree, Markov processes) as compared to dynamic models (e.g. system dynamics, individual-based models). In dynamic models, the risk of infection can change over time. There has been no apparent increase in the application of dynamic modelling and no relationship between modelling type and year of publication [80]. Although dynamic models are not always necessary to represent vaccine preventable diseases (e.g. tetanus, herpes zoster), they allow for the intrinsic consideration of vaccine externalities (herd immunity, shifts in age of infection and serotype replacement). Consideration of externalities is limited in the Canadian literature, with fewer than half of studies in our review considering herd immunity. Failure to include the impact of herd immunity can significantly underestimate the effectiveness of a vaccine [80]. An increase in dynamic modelling, where relevant, could improve the accuracy of economic evaluations of vaccines and should be an area of future research.

Individual-based models are, however, becoming increasingly popular in economic evaluations [21, 28, 30, 31, 70]. These models have the advantage of being able to replicate individual-level behaviour and interactions (e.g. transmission, risk behaviours, disease history), which may have substantial impacts on the economic evaluation. Individual-based models not only intrinsically account for the impact of herd immunity but also help researchers study the influence of key subsets of the population. Three separate economic evaluations on HPV used a common individual-based model called HPV-ADVISE to estimate the costs and benefits of different vaccination strategies and scenarios in Canada [28, 30, 31]. The HPV-ADVISE model informed HPV vaccine policy-making regarding the cost-effectiveness of various vaccine types (bivalent versus quadrivalent versus nine-valent), catch-up programs, type replacement, schedules (ages, number of doses), as well as male-vaccination. HPV-ADVISE is an example of the power and flexibility of individual-based modelling and how information sharing can improve economic evaluation research and therefore the policy-informing power of these types of studies. However, there are also trade-offs to consider with dynamic models, specifically individual-based models, as they are often more complex, time consuming and can require more fine grained data, potentially impacting the reliability and timeliness of the results.

Authors’ choice of the time horizon and discount rate can significantly impact the outcomes of an economic evaluation. As is noted in many guidelines for economic evaluations, for accurate analysis the chosen time horizons must encompass all costs and benefits of a policy decision [16]. However, as we saw in this analysis many economic evaluations adopt a shorter than necessary time horizon (e.g. economic evaluations adopting the same time horizon as a clinical trials), creating the possibility of a time horizon bias. Another important element of economic evaluations that can significantly impact the outcomes of a cost-effectiveness analysis is the perspective and therefore, the consideration of costs and benefits not directly related to health. It is essential that economic evaluations clearly justify their choice of perspective and discuss the costs not considered, however a complete discussion of choice of perspective was often missing in the studies of our analysis. Studies are starting to adopt a two -perspective approach, where they not only consider the economic benefits and costs from a narrower view point (e.g. healthcare payer) but also using the most comprehensive approach (i.e. societal perspective). Reporting of time horizon and study perspectives in economic evaluation of vaccines must improve to ensure the transparency of complex analyses and to provide the details necessary for informed decision-making.

In our review only six articles included some assessment or discussion of equity in their analysis. Lack of equity considerations and inability to account for equality in resource allocation analyses are common criticisms of economic evaluations [81]. Debate continues around whether or not economic evaluations have the responsibility to consider equity; however, the lack of information on the differential impact of interventions often mean policy-makers are hesitant to use the results of economic evaluations [81]. In recent years there have been a number of methodological advancements to help economists account for equity as part of, or as an extension to, their cost-effectiveness analysis (e.g. extended or distributional cost-effectiveness); however, the use of these tools is still limited [82]. As the field of economic evaluations of vaccines continues to grow it is important that these studies consider and discuss how their findings may have an impact on the equity of health care delivery and health outcomes.

Research shows that guidelines for economic evaluations from non-governmental organizations, governments and journals can help increase the transparency and reliability of these studies [77]. In fact, one of the main reasons the WHO created guidelines for economic evaluations of vaccines in 2008 [3] was to address the limitations they observed in evaluations reported prior to 2005, and to enhance standardization and comparability between studies [2]. Both Baladi et al. and Neumann et al. discovered a growing adherence to recommended practices in cost-effectiveness and cost-utility analyses worldwide and in Canada, which they attributed to stricter journal protocols and guidelines in publishing these studies [77, 83]. Our results further support these findings indicating that since the publication of WHO and CADTH guidelines there has been an increase in the standardization of the methods used in economic evaluation and in the transparency and reporting of the research in Canada [3, 16]. Although, we noted a change in the consistency of reporting following the introduction of the guidelines, there remained certain study elements commonly omitted, such as the study perspective, modelling technique description and time-horizon. The large number of assumptions and parameters used in economic evaluations make reporting guidelines essential, as simple changes to the perspective adopted, discount rate or time horizon chosen can have a major impact on the results [6]. Therefore, the transparency and reliability of these studies should continue to improve and the guidelines must be continually updated to ensure that the results are both useable and translatable.

The use of national guidelines and publishing protocols is particularly relevant and important to our sample of articles, as just under half the studies had reported conflicts of interest and these studies were significantly more likely to find the vaccine of interest cost-effective. Therefore, transparency and consistency in economic evaluations are essential to ensure policy-makers can use the results to make accurate and unbiased decisions.

This study had a number of limitations. First, we were unable to include articles in French, and therefore may have missed contributions to the economic evaluation literature, especially as Quebec was the most prolific region in the production of economic evaluations of vaccines. Second, only one reviewer was able to screen the grey literature articles for inclusion. Nevertheless, all final decisions were discussed between the two reviewers to ensure agreement about all studies included in the analysis. Finally, we did not compare the results of the economic evaluations because we had included articles for a variety of diseases and with a wide range of quality.

## Conclusions

To date economic evaluations of vaccines in Canada have not been summarised or reviewed, potentially limiting the influence these studies have on policy-making. This scoping review acts as a guiding document for policy-makers and researchers, to provide easy access to essential information about vaccines. Our scoping review outlined many gaps in the literature, including the lack of studies on various privately-available vaccines, different vaccination schedules and programs, and the shortage of ex-post implementation economic evaluations. Furthermore, we identified important trends in the literature, including an increasing number of economic evaluations on vaccines and a focus on newly-available vaccines.

We found some weaknesses in the literature, including the limited use of dynamic modelling and consideration of herd immunity, the significant association between declared conflicts of interest and a positive cost-effectiveness result, as well as the under-reporting of time-horizon, perspective and modelling type. However, the scoping review outlined some key strengths of the Canadian literature, including an increase in the application of individual-based modelling techniques. Furthermore, the implementation of national guidelines appears to have had an impact on the standardization and transparency of economic evaluations in Canada, and remains an important consideration for countries without similar guidelines. As more countries start to map this literature and analyse the trends and limitations, then comparisons between countries, their economic evaluation literature and their vaccination programs, could provide valuable input for improving the quality and usefulness of economic evaluations of vaccines.

## Additional file

**Additional file 1: Table S1.** Keyword search strategy to identify peer-reviewed articles. This table provides a description of the search terms we used in our review.

## Abbreviation

HPV: human papillomavirus
