# Supplementary material for: Economic evaluations of vaccines in Canada: a scoping review
Source: Cost Eff Resour Alloc. 2017 May 5;15:7. doi: 10.1186/s12962-017-0069-4 (PMC5420143; doi:10.1186/s12962-017-0069-4)
Supplement: Supplementary file 1 — Additional file 1: Table S1. Keyword search strategy to identify peer-reviewed articles. This table provides a description of the search terms we used in our review. [file 12962_2017_69_MOESM1_ESM.docx]

Additional File 1

**Table 1: Keyword search strategy to identify peer-reviewed articles**

| **Immunization keywords** | **Economic evaluation keywords** | **Canadian keywords** |
| --- | --- | --- |
| Immunization  Immunizations  Immunisation  Immunisations  Immunized  Immuni* program  Immuni* programs  Immuni* schedule  Immunization, Secondary  Mass Immuni*  Vaccine  Vaccines  Vaccination  Vaccinations  Vaccinating  Mass vaccination  Vaccin* program  Vaccin* programs | Economic evaluation  Economic evaluations  Cost benefit  Cost effective  Cost effectiveness  Cost utility  Cost*  Cost benefit analysis  Cost benefit analyses  Cost effectiveness analysis  Incremental cost effectiveness  Incremental cost utility  Incremental economic evaluation  Models, economic  Cost saving*  Cost of illness  Cost and cost analysis  Cost minimization | Canada  British Columbia*  Alberta*  Saskatchewan*  Manitoba*  Ontario*  Quebec*  Maritimes  New Brunswick  Nova Scotia  Prince Edward Island  PEI  Northwest Territories  Nunavut  Yukon  Toront*  Calgar*  Edmonton*  Vancouver*  Winnipeg*  Ottawa*  Halifax*  Saskatoon*  Regina  Victoria |
